# Serum lactate is an independent predictor of hospital mortality in critically ill patients in the emergency department: a retrospective study

**DOI:** 10.1186/s13049-017-0415-8

**Published:** 2017-07-14

**Authors:** Ralphe Bou Chebl, Christopher El Khuri, Ali Shami, Eva Rajha, Nagham Faris, Rana Bachir, Gilbert Abou Dagher

**Affiliations:** 1 Department of Emergency Medicine, American University of Beirut Medical Center, Beirut, Lebanon; 2 Department of Emergency Medicine, Baylor College of Medicine, Houston, TX USA

**Keywords:** Lactate, Lactic acid, Emergency, Mortality

## Abstract

**Background:**

Elevated lactate has been found to be associated with a higher mortality in a diverse patient population. The aim of the study is to investigate if initial serum lactate level is independently associated with hospital mortality for critically ill patients presenting to the Emergency Department.

**Methods:**

Single-center, retrospective study at a tertiary care hospital looking at patients who presented to the Emergency Department (ED) between 2014 and 2016. A total of 450 patients were included in the study. Patients were stratified to lactate levels: <2 mmol/L, 2-4 mmol/L and >4 mmol/L. The primary outcome was in-hospital mortality. Secondary outcomes included 72-h hospital mortality, ED and hospital lengths of stay.

**Results:**

The mean age was 64.87 ± 18.08 years in the <2 mmol/L group, 68.51 ± 18.01 years in the 2-4 mmol/L group, and 67.46 ± 17.67 years in the >4 mmol/L group. All 3 groups were comparable in terms of age, gender and comorbidities except for diabetes, with the 2-4 mmol/L and >4 mmol/L groups having a higher proportion of diabetic patients. The mean lactate level was 1.42 ± 0.38 (<2 mmol/L), 2.72 ± 0.55 (2-4 mmol/L) and 7.18 ± 3.42 (>4 mmol/L). In-hospital mortality was found to be 4 (2.7%), 18(12%) and 61(40.7%) patients in the low, intermediate and high lactate groups respectively. ED and hospital length of stay were longer for the >4 mmol/L group as compared to the other groups. While adjusting for all variables, patients with intermediate and high lactate had 7.13 (CI 95% 2.22–22.87 *p* = 0.001) and 29.48 (CI 95% 9.75–89.07 *p* = <0.001) greater odds of in-hospital mortality respectively.

**Discussion:**

Our results showed that for all patients presenting to the ED, a rising lactate value is associated with a higher mortality. This pattern was similar regardless of patients’ age, presence of infection or blood pressure at presentation.

**Conclusion:**

Higher lactate values are associated with higher hospital mortalities and longer ED and hospital lengths of stays. Initial ED lactate is a useful test to risk-stratify critically ill patients presenting to the ED.

## Background

Lactate is the end product of pyruvate metabolism via the enzyme lactate dehydrogenase [1, 2]. Elevated Lactate levels have been associated with increased morbidity and mortality in a diverse patient population including: trauma, sepsis, multiple organ failure and elderly patients [3–10]. Additionally, lactate levels have been used to risk stratify septic patients to determine their disposition and intensity of treatment. Septic patients typically would be stratified to <2 mmol/L (low level) or >4 mmol/L (high level) with one of the treatment end points aimed at lowering serum lactate levels [11–13]. However, few studies have looked at lactate’s role as an independent predictor of mortality in patients presenting to the emergency department (ED) [10, 14]. The aim of this study was to ascertain if initial serum lactate level is independently associated with in-hospital mortality among critically ill patients presenting to the emergency department (ED).

## Methods

### Study design and setting

This was a single center, retrospective, chart review, cohort study conducted in an academic ED of a large tertiary care center. The study was approved by the American University of Beirut’s institutional review board (AUB IRB ER.RB.01). All patients presenting to the ED from January 2014 to January 2016 had their medical records queried via the hospital’s Electronic Health Record (EHR). Research assistants extracted all information from scanned charts and electronic laboratory reports. Before the initiation of data collection, multiple meetings with the principle investigators were conducted to standardize the process.

The inclusion criteria were: patients ≥18 years of age, an emergency severity index (ESI) level of ≤3 at presentation, required hospital admission and an initial serum lactate level drawn in the ED.

#### Sample size calculation

After the pooling of all patients that fit our criteria, patients were stratified into 3 groups: those with a serum lactate of <2 mmol/L (low level), 2-4 mmol/L (intermediate level) and >4 mmol/L (high level). Power calculation assumed a hospital mortality of patients with lactate levels of <2 mmol/L, 2-4 mmol/L and >4 mmol/L to be 4%, 11% and 23% respectively from an extensive literature review [11–13, 15]. In order to attain a power of 0.8 and an alpha of 0.05 for finding a difference in hospital mortality between low and intermediate levels in addition to intermediate and high levels of lactate, a minimum of 150 patients were required in each arm. The required number of patients was chosen from each group via a computer-randomized selection.

#### Exposure: serum lactate

Initial serum lactate is drawn in our ED by registered nurses before the initiation of resuscitation. Lactate tubes (lithium heparinized green top) are placed immediately on ice and delivered to the chemistry laboratory within 15 min for analysis. During the ED encounters, the indications for lactate testing included risk stratification of critically ill patients including suspected sepsis, trauma, and patients presenting with shock.

#### Outcome measures

The primary outcome of the study was in-hospital mortality. Secondary outcomes included: 72-h mortality as well as ED and hospital lengths of stay.

#### Other factors

The exposure of interest was initial lactate level. Control variables that were collected included: Patients’ age, gender, past medical history, recent surgical history, pregnancy status and use of lactate elevating medications (metformin, isoniazid, anti-retroviral drugs). Discharge diagnoses were noted from the patients’ discharge summaries and ICD-9 coding. Vital signs and laboratory tests were also obtained. Additionally, patient disposition; either discharged, or admitted to the intensive care unit (ICU) or regular floor was documented.

#### Statistical analysis

Statistical analyses were performed using SPSS 23.0 (SPSS Inc., Chicago, IL, USA). The distributions of the continuous and categorical variables were presented as mean ± standard deviation and frequency/percentages respectively. Stratifying selected characteristics by hospital mortality and by lactate level was done using the Pearson’s chi-square test/Fischer test and the one-way ANOVA where applicable. Tests were interpreted at a significance level (alpha ≤0.05).

In the bivariate analysis, Student’s t-test and Pearson’s chi square test were used to assess the significance of the statistical association between the independent variables (continuous and categorical) and hospital mortality; the dependent variable. Both tests were interpreted at a predetermined significance level (alpha = 0.05). Furthermore, the magnitude of association between the predictor variables and hospital mortality was determined through the calculation of odds ratios (OR) and their corresponding 95% confidence intervals (CI). A multivariate analysis was performed using logistic regression to look for the association between the primary outcome variable and all predictor factors. A backward selection procedure, with significance level for removal from the model set at 0.1, was conducted by fitting hospital mortality with all risk factors found to be significant in the bivariate level, in addition to those considered as being clinically meaningful. Furthermore, a goodness of fit was done to assess if the final model was good enough at discriminating between the two categories of the outcome variable “hospital mortality: yes/no”.

## Results

### Population characteristics

A total of 450 patients were included in the study with 150 allocated to each lactate group of <2 mmol/L, 2-4 mmol/L and >4 mmol/L. The mean age of the patients was 64.87 ± 18.08 years in the <2 mmol/L group, 68.51 ± 18.01 years in the 2-4 mmol/L group, and 67.46 ± 17.67 in the >4 mmol/L group. All 3 groups were comparable in terms of age, gender and comorbidities except for diabetes with the 2-4 mmol/L and >4 mmol/L groups having a higher proportion of diabetic patients. Metformin use was more prevalent in these two groups as well. None of the study patients were found to be taking Isoniazid or anti-retroviral drugs. The most common discharge diagnosis in all strata was infection related (79.3, 84.7 and 76.7% respectively). The information is summarized in Table 1.

**Table 1:** Population characteristics

	<2 mmol/L (*N* = 150)	2–4 mmol/L (*N* = 150)	>4 mmol/L (*N* = 150)	*p*-value
Age (years) (Mean ± SD)	64.87 ± 18.08	68.51 ± 18.01	67.46 ± 17.67	0.194
Male sex no.(%)	78 (52.0)	92 (61.3)	86 (57.3)	0.262
DM no.(%)	43 (28.7)	67 (44.7)	77 (51.3)	<0.001
CAD no.(%)	44 (29.3)	48 (32.0)	58 (38.7)	0.210
CKD no.(%)	32 (21.3)	27 (18.0)	34 (22.7)	0.589
Metabolic illness no.(%)	5 (3.3)	0 (0.0)	2 (1.3)	0.063
Malignancy no.(%)	50 (33.3)	34 (22.7)	45 (30.0)	0.113
Recent surgery no.(%)	3 (2.0)	4 (2.7)	9 (6.0)	0.134
Medication use no.(%)				
Metformin	9 (6.0)	29 (19.3)	27 (18.0)	0.001
Discharge diagnosis no.(%)				
Infection	119 (79.3)	127 (84.7)	115 (76.7)	0.208
Trauma	5 (3.3)	7 (4.7)	13 (8.7)	0.111
Cardiogenic shock	0 (0.0)	0 (0.0)	27 (18.0)	<0.001
MI	1 (0.7)	2 (1.3)	10 (6.7)	0.003
Stroke	4 (2.7)	3 (2.0)	4 (2.7)	0.911
Neurogenic shock	0 (0.0)	0 (0.0)	3 (2.0)	0.049
DKA	0 (0.0)	2 (1.3)	4 (2.7)	0.132
Ethanol toxicity	1 (0.7)	2 (1.3)	1 (0.7)	0.777
Acute limb ischemia	3 (2.0)	2 (1.3)	3 (2.0)	0.881
DVT	0 (0.0)	0 (0.0)	3 (2.0)	0.049
DIC	0 (0.0)	0 (0.0)	2 (1.3)	0.134
Obstetric emergencies^a^	2 (1.3)	0 (0.0)	0 (0.0)	0.134

^a^Diagnoses included: PPROM (Preterm premature rupture of membranes) and Missed Abortion

### Vital Signs and laboratory tests

Table 2 presents the vital signs and laboratory results upon admission to the ED. Heart rate (HR), systolic blood pressure (SBP), temperature, lactate level and white blood cell (WBC) count differed significantly among the 3 strata. The mean initial lactate level in each group was 1.42 ± 0.38 (<2 mmol/L), 2.72 ± 0.55 (2-4 mmol/L) and 7.18 ± 3.42 (>4 mmol/L). Arterial pH was also found to be different in the >4 mmol/L group with mean pH of 7.31 ± 0.16; lower than the other 2 groups (7.38 ± 0.09 and 7.35 ± 0.09 for the 2-4 mmol/L and <2 mmol/L groups respectively). SBP was found to be 120.03 ± 28.23 in the low lactate group, 124.56 ± 28.54 in the intermediate lactate group and 114.13 ± 29.92 in the high lactate group. Temperature was lower in the >4 mmol/L lactate group (37.05 ± 1.10) when compared to the low and intermediate groups (37.23 ± 1.04 and 37.42 ± 1.04 respectively). HR and WBC trended higher with increasing lactate levels.

**Table 2:** Vital signs and laboratory tests upon presentation

	<2 mmol/L (*N* = 150)	2–4 mmol/L (*N* = 150)	>4 mmol/L (*N* = 150)	*p*-value
HR (bpm) (Mean ± SD)	95.22 ± 21.27	100.69 ± 24.14	103.59 ± 23.74	0.007
RR (Breaths/min) (Mean ± SD)	21.91 ± 5.54	22.09 ± 5.54	23.22 ± 6.11	0.110
SBP (mmHg) (Mean ± SD)	120.03 ± 28.23	124.56 ± 28.54	114.13 ± 29.92	0.009
DBP (mmHg) (Mean ± SD)	66.31 ± 17.88	67.31 ± 18.24	65.12 ± 22.06	0.626
O_2_ saturation (%) (Mean ± SD)	95.09 ± 7.83	95.03 ± 9.28	93.37 ± 8.30	0.147
Temperature (°C) (Mean ± SD)	37.23 ± 1.04	37.42 ± 1.04	37.05 ± 1.10	0.013
Lactate level (mmol/L) (Mean ± SD)	1.42 ± 0.38	2.72 ± 0.55	7.18 ± 3.42	<0.001
WBC (x10^9^cells/L) (Mean ± SD)	11,071.33 ± 6908.81	12,728.00 ± 6860.88	15,583.33 ± 12,135.99	<0.001
Hemoglobin (mg/dl) (Mean ± SD)	11.54 ± 3.37	11.58 ± 2.19	11.30 ± 2.56	0.640
Hematocrit (mg/dl) (Mean ± SD)	33.46 ± 7.10	34.69 ± 6.55	34.30 ± 8.10	0.331
Arterial pH (Mean ± SD)^a^	7.35 ± 0.09	7.38 ± 0.09	7.31 ± 0.16	0.001
PaCO_2_ (mmHg) (Mean ± SD)^a^	41.63 ± 21.57	37.35 ± 13.22	34.98 ± 16.22	0.098
PaO_2_ (mmHg) (Mean ± SD)^a^	97.03 ± 47.74	97.45 ± 48.05	109.53 ± 70.75	0.350
Troponins (mg/dl) (Mean ± SD)^b^	0.10 ± 0.22	0.07 ± 0.12	0.11 ± 0.13	0.254
Creatinine (mg/dl) (Mean ± SD)	1.63 ± 1.94	1.44 ± 1.35	1.90 ± 1.65	0.053

^a^Obtained from 205 patients: 41 (<2 mmol/L), 64 (2–3.9 mmol/L) and 100 (>4 mmol/L)

^b^Obtained from 158 patients: 39 (<2 mmol/L), 56 (2-4 mmol/L) and 63 (>4 mmol/L)

### Length of stay and in-hospital mortality analysis

As shown in Table 3, length of stay in the ED was found to be different among the 3 strata with patients in the >4 mmol/L lactate cohort staying 16.49 ± 16.83 h as opposed to 11.83 ± 21.05 for the <2 mmol/L group and 11.71 ± 13.04 for the 2-4 mmol/L group (*p* = 0.025). Patients with a lactate >4 mmol/L also had a slightly longer hospital length of stay as compared to the other two groups. The mean hospital LOS was found to be 10.36 ± 12.60 days in the lactate >4 mmol/L group as compared to 8.09 ± 8.78 days for the 2-4 mmol/L group and 7.80 ± 8.06 in the <2 mmol/L group. Out of all the patients in their respective groups, 4 (2.7%), 18 (12%) and 61 (40.7%) died within their hospital stay (*p* = <0.001). There were no 72-h mortalities in the low and intermediate lactate level groups in contrast to 25 (16.6%) in the high lactate group (*p* = <0.001).

**Table 3:** Mortality and length of stay

	<2 mmol/L (*N* = 150)	2–4 mmol/L (*N* = 150)	>4 mmol/L (*N* = 150)	*p*-value
Length of stay ED (hours) (Mean ± SD)	11.83 ± 21.05	11.71 ± 13.04	16.49 ± 16.83	0.025
Length of stay Hospital (days) (Mean ± SD)	7.80 ± 8.06	8.09 ± 8.78	10.36 ± 12.60	0.055
Mortality no.(%)				
Hospital	4 (2.7)	18 (12.0)	61 (40.7)	<0.001
72-h	0 (0.0)	0 (0.0)	25 (16.7)	<0.001

In-hospital mortalities were stratified according to age (<65 years and ≥65 years), systolic blood pressure (<90 mmHg and ≥90 mmHg) and infection status (Yes/No) for each lactate subgroup. It was noted that, regardless of age, infection status or blood pressure, there was an increase in in-hospital mortality with an increasing lactate level. These results are summarized in Table 4.

**Table 4:** In-hospital mortality among the different lactate groups stratified to ages ≥65 and <65 yrs., infected/non-Infected and SBP <90 mmHg and ≥90 mmHg

Hospital mortality					
Lactate		<2 mmol/L (*N* = 150)	2-4 mmol/L (*N* = 150)	>4 mmol/L (*N* = 150)	*P*-value
Strata no.(%)					
Age (years)	<65	2 (3.1)	3 (6.8)	18 (36.0)	<0.001
	≥65	2 (2.3)	15 (14.2)	43 (43.0)	<0.001
Infection	Yes	2 (1.7)	17 (13.4)	47 (40.9)	<0.001
	No	2 (6.5)	1 (4.3)	14 (40.0)	<0.001
SBP (mmHg)	SBP < 90	3 (15.0)	2 (11.1)	10 (35.7)	0.092
	SBP ≥ 90	1 (0.8)	16 (12.1)	44 (38.3)	<0.001
Total		4 (2.7)	18 (12.0)	61 (40.7)	<0.001

Furthermore, a subgroup analysis was done by stratifying each lactate group by infection status, age and blood pressure in order to look for associations between these variables in regards to hospital mortality. Although the results were not statistically significant, patients older than 65 years had a higher mortality than patients younger than 65 years of age (15(14.2%) versus 3(6.8%) and 43 (43.0%) versus 18 (36.0%) respectively) in the intermediate and the high lactate groups. In a similar manner, patients with an infectious process as a cause for their lactate elevation had a higher in-hospital mortality compared to patients who did not have any infection (17(13.4%) versus 1 (4.3%) in the intermediate group. The mortality was similar in the high lactate group regardless of infection (47 (40.9%) versus 14 (40%)). Absence of infection was associated with a higher mortality in the low lactate group (2 (6.5%) versus 2 (1.7%)). Finally, normotensive patients were found to have a slightly higher mortality in the intermediate and high lactate groups when compared to hypotensive patients. In contrast, in the low lactate cohort, hypotensive patients had a higher mortality than normotensive patients Table 5.

**Table 5:** Sub-analysis stratification of lactate levels by infection status, blood pressure and age in terms of hospital mortality

Lactate Group	Infection Status	Hospital Mortality	*p*-value
< 2	No Yes	2 (6.5%) 2 (1.7%)	0.142
2–4	No Yes	1 (4.3%) 17 (13.4%)	0.220
> 4	No Yes	14 (40.0%) 47 (40.9%)	0.927
Lactate Group	SBP	Hospital Mortality	*p*-value
< 2	<90 ≥90	3 (15.0%) 1 (0.8%)	<0.001
2–4	<90 ≥90	2 (11.1%) 16 (12.1%)	0.902
> 4	<90 ≥90	10 (35.7%) 44 (38.3%)	0.803
Lactate group	Age	Hospital Mortality	*p*-value
< 2	<65 ≥65	2 (3.1%) 2 (2.3%)	0.764
2–4	<65 ≥65	3 (6.8%) 15 (14.2%)	0.208
> 4	<65 ≥65	18 (36.0%) 43 (43.0%)	0.411

Table 6 shows the multiple logistic regression that was performed in order to ascertain the independent association between lactate and in-hospital mortality. The <2 mmol/L group was taken as a reference group during the multivariate analysis. The unadjusted ORs were 4.97 (CI95% 1.64–15.08) for the intermediate group and 25.02 (8.79–71.16) for the high lactate group. When adjusted for all statistically significant and clinically meaningful variables in the bivariate analysis, patients with intermediate and high lactate levels had a 7.13 (2.22–22.87 *p* = 0.001) and 29.48 (9.75–89.07 *p* = <0.001) increased odds of in-hospital mortality respectively. Furthermore, a goodness of fit test (*p* = 0.147) and ROC curve (A = 0.834 *p* = <0.001) indicated that the final model was good enough at discriminating between the two categories of the outcome variable “hospital mortality: yes/no”.

**Table 6:** Multiple Logistic Regression for in-hospital mortality

	Crude^a^		Adjusted^b^	
	OR (Cl 95%)	*p*-value	OR (Cl 95%)	*p*-value
2-4 mmol/L	4.97 (1.64–15.08)	0.005	7.13 (2.22–22.87)	0.001
>4 mmol/L	25.02 (8.79–71.16)	<0.001	29.48 (9.75–89.07)	<0.001

^a^Reference group: lactate group <2 mmol/L

^b^While controlling for all statistically significant and clinically relevant variables from the bivariate analysis in Table 1

## Discussion

In states of tissue hypo-perfusion, the fall in oxygen delivery causes a shift to an anaerobic glycolysis where the primary energy source for cells is provided by pyruvate’s conversion into lactate. This ultimately leads to a rise in the serum lactate level, which normally, ranges between 1 and 2 mmol/L. [1, 2, 16, 17] Elevated lactate levels have been associated with poor outcomes in septic patients. Following the publication of the EGDT trial, lactate measurement was incorporated by the surviving sepsis campaign (SSC) as part of the initial management of patients with a suspected infection [11, 15]. According to the SSC, a lactate of >4 mmol/L in a septic patient warrants aggressive therapy. However, there were recent studies that showed that infected patients with a lactate between 2 and 4 mmol/L have a risk of mortality that is twice that of patients with a lactate level < 2 mmol/L. [8–13, 15] In our study, we aimed to evaluate the independent association between initial serum and in-hospital mortality for critically ill patients who presented to the emergency department.

Our results showed that for all patients presenting to the ED, a rising lactate value is associated with a higher mortality. Our findings are in keeping with the current literature describing the predictive ability of initial lactate levels on in-hospital mortality [17–19], as we were able to show that patients with a lactate of 2-4 mmol/l had an in-hospital mortality of 12% and more importantly, patients with a lactate >4 mmol/L were found to have an in-hospital mortality of 40.7%. Soliman et al. studied the association between lactate elevation and increased hospital length of stay. They looked at patients admitted to their ICU and noticed that patients with elevated lactate who survived to discharge had a longer length of stay than patients with normal lactate levels [20]. Our results were similar to the literature, as we found that the ED and hospital lengths of stay were longer as the lactate level increased.

Furthermore, several studies have independently tried to look at the prognostic value of lactate in the setting of an infectious source, in normotensive patients, and finally in elderly patients. Shapiro et al. looked at 1278 patients admitted to their hospital for an infection related cause. According to their study, mortality rates increased as lactate increased: 43 (4.9%) of 877 of patients with a lactate level between 0 and 2.5 mmol/L died, 24 (9.0%) of 267 patients with a lactate level between 2.5 and 4.0 mmol/L died, and 38 (28.4%) of 134 patients with a lactate level greater than or equal to 4.0 mmol/L died [21]. We noticed a similar trend in our patient cohort. As the lactate level rose in infected patients, mortality increased from 1.7% (<2 mmol/L group) to 13.4% (2-4 mmol/L group) to 40.9% (>4 mmol/L group). While our findings are in tune with Shapiro et al. it is important to note that patients with a lactate >4 mmol/L presenting without an infectious etiology still had a very high in-hospital mortality (40%). Additionally, there was no statistically significant difference in mortality between infected and non-infected patients. This further points out that the prognostic ability of lactate seems independent of the presence of infection.

Infected patients who present to the ED hypotensive and with an elevated lactate have poor outcomes, with mortalities ranging from 23 to 40% [11–13, 15]. Howell et al. looked at the role of lactate in patients with a suspected infection and were able to show that elevated lactate was associated with increased mortality regardless of hypotension [22]. In our study we stratified our patients according to SBP at presentation to the ED and compared hypotensive (<90 mmHg) to normotensive (≥90 mmHg) patients among each lactate cohort and found that there was a slight increase in death with normotension except for the low lactate group. This is probably due to the fact that hypotensive patients receive more aggressive care early on in the emergency department, as opposed to patients who presented with normal blood pressure. Arnold RC et al., who showed that normotensive adults with suspected infection and elevated lactate had increased organ dysfunction and received relatively little IV fluid therapy during ED encounters, also noted this finding [23].

Moreover, a recent study by Del Portal et al. looked at the prognostic role of lactate in elderly patients and examined its association with mortality. They looked at patients older than 65 years of age presenting to the emergency department and stratified them according to presence or absence of infection and found that increasing lactate levels are associated with greater mortality in this subset of patients regardless of the presence or absence of infection [10]. Our results were similar to Del portal et al. as we noticed an increased mortality amongst elderly patients. However, it is important to note that when examining patients <65 years of age, the in-hospital mortality increased significantly as the lactate level increased.

Lactate testing in the ED seems to be a valid prognostic marker of in-hospital mortality. While it seems to be of more use in the elderly patients, a lactate level > 4 mmol/L carries a poor prognosis in critically ill patients. In their research study, Trzeciak et al. concluded that an initial lactate ≥4.0 mmol/l was associated with six-fold higher odds of acute-phase death. Furthermore, according to Howell et al. a lactate ≥4.0 mmol/l had 7.1 times the odds of death than patients with a normal lactate. In a similar manner, and after adjusting for all the variables, lactate elevation in our study was associated with an increase in in-hospital mortality. A lactate level > 2 was associated with a 7.1 times the odds of death and a lactate level > 4 was associated with 29.4 times the odds of death than patients with normal lactate. Our findings further validate the role of serum lactate as a risk-stratifying test and a prognostic tool in the emergency department.

### Limitations

This was a retrospective chart review cohort study and the authors are aware of the inherent limitations with possible misclassification and ascertainment bias. Additionally, ICD-9 coding/discharge diagnoses errors may occur with the electronic health record chart review process. To minimize these biases, regular meetings with the PI of the study were conducted to standardize interpretation and collection of the information.

The study attempted to encompass a more diverse patient population in regards to the effect of lactate but due to the nature of the study and the choice of patients who had already had their lactate taken; the population was comprised mostly of those diagnosed with infection: a population whose lactate levels hold a known correlation with mortality and morbidity. However, our sub-analysis showed regardless of lactate level that infection status did not affect mortality. It should be noted that these infected patients all received protocol-based sepsis treatment thus all received the same standard of care.

The noted mortalities in each lactate group in this study are higher than those noted in the literature. This can be explained by our choice of critically ill patients in addition to the possible selection bias whereby patients that have their lactate drawn are more likely to have a more severe disease process and therefore are results cannot be generalized to the general population. Though baseline mortalities were higher, our results still showed a consistent increasing trend in-hospital mortality with increasing lactate levels.

Finally, the cohorts chosen were not matched for comparison possibly limiting our drawn conclusions about mortality difference among them. This was accounted for by performing a multivariate analysis to control for differences between the groups and all clinically meaningful variables. Additionally, descriptive analysis showed that the 3 groups were comparable among most of their demographics.

## Conclusion

Serum lactate is an important prognostic marker of in-hospital mortality. Initial lactate in the ED is a useful test to risk-stratify critically ill patients in the ED regardless of age, presenting vital signs or presence of infection. It may also be useful in determining patients’ disposition and level of care. We hope that this study stimulates further research on the value of lactate as a prognostic and screening laboratory test in the Emergency Department.

## Abbreviations

CAD: Coronary Artery Disease
CKD: Chronic Kidney Disease
DBP: Diastolic Blood Pressure
DIC: Disseminated Intravascular Coagulation
DKA: Diabetic Ketoacidosis
DM: Diabetes Mellitus
DVT: Deep Vein Thrombosis
ED: Emergency Department
HR: Heart Rate
LOS: Length of Stay
MI: Myocardial Infarction
RR: Respiratory Rate
SBP: Systolic Blood Pressure
WBC: White Blood Cell
